# Associations between the universal health coverage service coverage index and the human development index: A country-level longitudinal panel study

**DOI:** 10.1371/journal.pgph.0007328

**Published:** 2026-10-28

**Authors:** Akira Hayasaka, Yasuhiro Nakanishi, Marika Nomura, Eri Osawa

**Affiliations:** 1 Graduate School of Data Science, Yokohama City University, Yokohama, Kanagawa, Japan; 2 Department of Health and Welfare Services, National Institute of Public Health, Wako, Saitama, Japan; 3 Human Development Department, Japan International Cooperation Agency (JICA), Chiyoda-ku, Tokyo, Japan; 4 School of Tropical Medicine and Global Health, Nagasaki University, Nagasaki, Japan; 5 Department of Public Health Policy, National Institute of Public Health, Wako, Saitama, Japan; University of Milano–Bicocca: Universita degli Studi di Milano-Bicocca, ITALY

## Abstract

Universal health coverage (UHC) and human development are closely linked, but whether this relationship differs across UHC domains remains unclear. We conducted a retrospective country-level longitudinal panel study using publicly available data from 2000 to 2021 to examine associations of the UHC service coverage index (SCI) and its four sub-indices with the Human Development Index (HDI) and its component indices for gross national income (GNI), education, and life expectancy. We also quantified differences in the UHC SCI, its sub-indices, and the HDI across World Bank income groups and World Health Organization regions using population-averaged generalized estimating equation models. UHC service coverage improved over time across income groups and regions, but progress was uneven. Persistent income-group disparities were observed for the overall UHC SCI, infectious diseases (IDs), service capacity and access (SCA), and the HDI, whereas disparities were smaller for reproductive, maternal, newborn, and child health (RMNCH) and less pronounced for noncommunicable diseases (NCDs). After covariate adjustment, regional differences were generally smaller than income-group disparities; RMNCH showed little regional variation, whereas SCA remained significantly lower in the African Region. Higher HDI was associated with better UHC performance, with adjusted increases of 6.8 points (95% CI 5.6–8.1) for the UHC SCI, 11.7 (95% CI 9.2–14.1) for SCA, 6.2 (95% CI 4.2–8.1) for ID, and 4.9 (95% CI 3.2–6.7) for RMNCH per 0.1-unit increase, whereas the increase for NCD was smaller (2.2 points, 95% CI 0.4–4.0). Among HDI components, the education index was not associated with NCD after adjustment, whereas the GNI and life expectancy indices were positively associated with NCD, although the estimated increases were smaller than those for most other sub-indices. These findings may help policymakers interpret the UHC SCI and its sub-indices in the context of their countries’ broader development trajectories.

## Introduction

Universal health coverage (UHC) is a core concept in global health policy and is positioned as target 3.8 of the Sustainable Development Goals (SDGs). To monitor progress toward UHC, the World Health Organization (WHO) and the World Bank developed the UHC service coverage index (SCI), a composite indicator for SDG 3.8.1. The overall SCI score for each country is calculated on a 0–100 scale as a geometric mean of four domain-specific sub-indices—reproductive, maternal, newborn, and child health (RMNCH); infectious diseases (IDs); noncommunicable diseases (NCDs); and service capacity and access (SCA). The four sub-indices are in turn constructed from 14 tracer indicators using geometric means, which is intended to prevent low coverage in one area from being fully masked by high coverage in another [1,2].

In 2025, as part of a comprehensive review of the SDG indicator framework, the United Nations Statistical Commission approved revisions to the SCI, including updates to three tracer indicators and the adoption of a population-weighted geometric mean approach [3,4]. The revised SCI retains the same name but is calculated differently, and these changes may affect both the levels and trends of UHC service coverage estimates [4]. Because pre-2025 SCI data series were used in this study, domain-specific findings should be interpreted in light of the tracer indicators used in that measurement framework, particularly for NCDs. In the 2023 WHO and World Bank monitoring framework, the NCD sub-index included hypertension treatment, diabetes prevalence, and tobacco non-use; therefore, it should be interpreted as a tracer-based summary measure rather than as a comprehensive measure of NCD service coverage, chronic disease management, or quality of NCD care [1].

Achieving UHC is considered closely linked to broader socioeconomic development. Countries with stronger economic capacity, higher educational attainment, better governance, and greater political stability tend to have stronger health systems and wider service coverage [5–7]. Conversely, improving health service coverage can improve population health and productivity, potentially supporting broader socioeconomic development [8]. Understanding the bidirectional relationship between UHC progress and human development can inform evidence-based health policymaking and help recognize the cross-sectoral value of investments in health service coverage.

In the foundational study reporting the establishment of the index, strong cross-sectional correlations were reported between the SCI and major global summary indicators of health and development, including the Human Development Index (HDI), under-five mortality, and life expectancy at birth [2]. These correlations were stronger than the correlation with gross national income (GNI) per capita alone, suggesting that the SCI captures dimensions of health system performance beyond economic prosperity. Subsequent studies have reported determinants of the SCI. A dominance analysis across 183 countries identified health workforce density as the most important overall predictor of the SCI, with several other determinants also contributing, although the relative importance of individual determinants varied by income group; for instance, in high-income countries, HIV treatment and cardiovascular disease-related indicators ranked higher than health workforce density [9]. A global panel analysis of 169 countries over 22 years found that higher per capita domestic health expenditure was significantly associated with improved SCI scores, but this effect was concentrated in the infectious disease domain and was weaker or non-significant for other domains such as RMNCH and NCDs [10]. Among low- and middle-income countries, governance quality and political stability have also been shown to be significant predictors of the SCI [6]. Other research has shown that higher SCI scores are associated with lower disease burden, as measured by disability-adjusted life years, with this association observed primarily for communicable, maternal, neonatal, and nutritional diseases, and little to no significant association for noncommunicable diseases or injuries [11]. Furthermore, the global monitoring report showed that progress in the SCI slowed during the SDG era, whereas improvements in the RMNCH and SCA sub-indices were more limited after 2015 [1].

Although existing research has deepened understanding of associations between the SCI and development, heterogeneity in the SCI–HDI association has not been systematically assessed at the global level using longitudinal data, particularly across income groups and WHO regions [2,12,13]. It also remains unclear whether the strength of the SCI–HDI association has changed over time as countries have followed different development trajectories. In addition, although the SCI is a composite measure built from four domain-specific sub-indices, associations of each sub-index with the HDI and its component indices (life expectancy, education, and GNI) have not been examined. Globally, the SCI and its four sub-indices follow different trajectories, and these trajectories also differ by income group [1].

Therefore, we aimed to analyze associations between the SCI and its four sub-indices and the HDI and its component indices using country-level longitudinal panel data. We quantified differences in the SCI, its sub-indices, and the HDI across World Bank income groups and WHO regions. We also assessed how each HDI component contributes to variation in the SCI and its sub-indices. These analyses provide context for interpreting the SCI and its sub-indices alongside broader development trajectories.

## Materials and methods

We conducted a retrospective, country-level longitudinal panel study using publicly available secondary data. The UHC SCI series analyzed in this study was obtained from the WHO Global Health Observatory [14] and was available only at seven benchmark years (2000, 2005, 2010, 2015, 2017, 2019, and 2021). We followed the STROBE reporting guideline [15].

### Ethics statement

Ethical approval was obtained from the Ethics Committee of the National Institute of Public Health (approval number: NIPH-IBRA#25010). We used publicly available country-level secondary data and included no individually identifiable information; therefore, informed consent was not required.

### Variables

The outcome variables were the UHC SCI and its four domain-specific sub-indices (0–100 scale) [14]. The key explanatory variables were the HDI and its component indices—life expectancy, education, and GNI—from the United Nations Development Programme Human Development Reports Data Center [16]. The HDI is calculated as the geometric mean of three normalized dimension indices capturing health (life expectancy at birth), education (mean years of schooling and expected years of schooling), and standard of living (GNI per capita, with income log-transformed) [17]. In analyses of the HDI components, the life expectancy index was included alongside the GNI and education indices because it is a formal component of the HDI. However, prior studies report that life expectancy may partly reflect downstream consequences of UHC progress; therefore, its temporal and causal interpretation in relation to the UHC SCI may differ from those of the GNI and education indices [8].

Countries were categorized into four World Bank income groups (low, lower-middle, upper-middle, and high income) using the World Bank Country and Lending Groups classification corresponding to each study year [18] and into six WHO regions (African Region (AFR), Region of the Americas (AMR), Eastern Mediterranean Region (EMR), European Region (EUR), South-East Asia Region (SEAR), and Western Pacific Region (WPR)) based on the WHO regional office classification [19]. Covariates included control of corruption from the Worldwide Governance Indicators [20,21] and demographic indicators from World Bank Open Data: population aged 65 years or older (% of the total population) [22], total fertility rate (births per woman) [23], and urban population (% of the total population) [24].

### Measures and data management

All datasets were reshaped into a long format at the country-year level and merged using country and study year as keys. HDI and its component indices were scaled from 0 to 1; to facilitate interpretability, effects were expressed per 0.1-unit increase in each index for the slope analyses. We used a complete-case analysis for each model, restricting estimation to observations with non-missing outcomes, explanatory variables, and covariates required for that specific model.

### Statistical analysis

We first summarized the distribution of outcomes, explanatory variables, grouping variables, and covariates by study year using country counts and median values with interquartile ranges (IQRs). We then described temporal patterns in the UHC outcomes and HDI by summarizing year-specific medians overall and within each World Bank income group and WHO region.

To estimate population-averaged differences by income group and WHO region while accounting for within-country correlations over time, we fitted generalized estimating equations (GEE) with country as the clustering unit [25,26]. We specified an independent working correlation structure and used robust (sandwich) variance estimators for inference because the UHC SCI and the four sub-indices used in this study were available only at seven benchmark time points with irregular intervals. For the UHC SCI, the four sub-indices, and the HDI, we used a Gaussian model with a log link and reported exponentiated coefficients as mean ratios with 95% confidence intervals (CIs). Unadjusted models included the main factor (income group or WHO region) and year fixed effects. Adjusted models additionally included covariates selected a priori to account for governance [5,6,12], demographic structure, population health needs, and service-access context [6,10]: control of corruption [20], population aged 65 years or older (%) [22], total fertility rate [23], and urban population (%) [24], with WHO region included in analyses by income group and income group included in analyses by WHO region. Health expenditure was not included in the primary adjustment set because, although it is an important correlate of UHC service coverage [5,6,10], it is closely linked to income and broader socioeconomic development and may represent a downstream financing mechanism through which public-sector capacity and policy priorities affect service coverage.

To quantify associations between human development and UHC outcomes as absolute differences on a 0–100 scale, we fitted GEE models with the UHC SCI and each sub-index as outcomes and HDI (or each component index) as the explanatory variable, using a Gaussian model with an identity link. We reported estimates as the expected change in outcome points per 0.1-unit increase in the index. We fitted models with adjustment only for year fixed effects and models additionally adjusted for control of corruption, population aged 65 years or older (%), total fertility rate, urban population (%), and WHO region. For all models, the working correlation structure was specified as independent, and robust standard errors and 95% CIs were computed.

Finally, in a supplementary analysis, we fitted interaction models allowing the index–outcome slope to vary by calendar year, including an interaction term between the index and year, with year modeled categorically. In these models, year-specific slopes were obtained from the interaction estimates and expressed per 0.1-unit increase in the index; the corresponding 95% CIs were computed by applying the same scaling to the standard errors. Robust (sandwich) variance estimation was used throughout the study.

All analyses and visualizations were conducted using R version 4.4.3 and RStudio version 2026.01.1 (Build 403). GEE models were fitted using the geepack package [27]. Statistical significance was assessed at a two-sided α of 0.05.

## Results

The UHC SCI and all four sub-indices had complete country coverage in each study year. In contrast, the HDI, its component indices, and control of corruption had more missing data—especially in the earlier years—resulting in year-to-year variation in the number of countries included in each analysis. When summarized by year using medians (IQR), the UHC SCI and most sub-indices showed clear improvements up to 2015, whereas progress largely plateaued thereafter, suggesting slower progress in overall service coverage in this dataset. Improvements were particularly pronounced for the ID sub-index, whereas RMNCH and NCD increased more modestly and appeared to level off after 2015; SCA increased earlier in the study period but declined somewhat in later years. The HDI and its component indices generally increased over time, with small setbacks in the most recent year. Control of corruption showed relatively little overall change over time, with only modest fluctuations in median values. Demographic covariates indicated gradual population aging and increasing urbanization alongside declining fertility (Table 1).

**Table 1:** Year-specific descriptive statistics for UHC, HDI, and covariates across countries in seven observation years.

Indicator	2000		2005		2010		2015		2017		2019		2021	
	n	Median (Q1, Q3)	n	Median (Q1, Q3)	n	Median (Q1, Q3)	n	Median (Q1, Q3)	n	Median (Q1, Q3)	n	Median (Q1, Q3)	n	Median (Q1, Q3)
UHC SCI	194	47.0 (28.0, 60.0)	194	54.0 (33.0, 67.0)	194	62.5 (42.5, 72.0)	194	69.0 (47.8, 77.0)	194	70.5 (51.2, 78.0)	194	70.5 (51.0, 79.0)	194	69.0 (52.0, 79.0)
RMNCH	194	71.0 (52.2, 81.8)	194	73.0 (54.2, 84.0)	194	75.0 (61.0, 84.0)	194	77.0 (63.0, 85.0)	194	76.0 (65.0, 85.0)	194	77.0 (64.0, 86.0)	194	76.0 (64.2, 86.0)
ID	194	18.5 (9.0, 39.0)	194	30.0 (14.0, 53.0)	194	50.0 (32.5, 65.8)	194	63.5 (45.0, 75.0)	194	69.0 (53.0, 79.0)	194	73.0 (55.2, 82.8)	194	68.0 (54.0, 83.0)
NCD	194	51.5 (43.2, 57.8)	194	55.0 (47.0, 61.0)	194	58.5 (50.0, 64.0)	194	60.0 (53.2, 66.0)	194	61.0 (54.0, 66.8)	194	62.0 (55.0, 67.0)	194	62.0 (55.2, 68.0)
SCA	194	69.5 (34.2, 87.0)	194	70.0 (34.2, 87.0)	194	71.0 (35.0, 87.0)	194	79.5 (41.2, 92.0)	194	79.0 (40.0, 92.0)	194	77.5 (38.0, 90.0)	194	75.0 (41.0, 89.8)
HDI	169	0.67 (0.50, 0.78)	183	0.69 (0.53, 0.79)	188	0.72 (0.56, 0.81)	189	0.74 (0.60, 0.84)	189	0.75 (0.61, 0.84)	189	0.75 (0.61, 0.85)	189	0.74 (0.61, 0.84)
GNI index	187	0.68 (0.53, 0.80)	188	0.70 (0.55, 0.83)	190	0.72 (0.57, 0.84)	190	0.74 (0.60, 0.86)	190	0.75 (0.61, 0.87)	190	0.76 (0.61, 0.88)	190	0.75 (0.61, 0.87)
Education index	171	0.57 (0.40, 0.69)	184	0.60 (0.44, 0.74)	188	0.65 (0.48, 0.77)	189	0.68 (0.53, 0.79)	189	0.69 (0.53, 0.80)	189	0.70 (0.54, 0.81)	189	0.71 (0.56, 0.82)
Life expectancy index	192	0.76 (0.61, 0.83)	192	0.78 (0.64, 0.85)	192	0.79 (0.67, 0.87)	192	0.80 (0.70, 0.89)	192	0.81 (0.70, 0.89)	192	0.81 (0.72, 0.89)	192	0.79 (0.70, 0.87)
Control of corruption	178	-0.25 (-0.87, 0.62)	188	-0.34 (-0.81, 0.49)	192	-0.34 (-0.78, 0.44)	192	-0.32 (-0.82, 0.61)	192	-0.29 (-0.79, 0.61)	192	-0.27 (-0.85, 0.60)	192	-0.28 (-0.83, 0.60)
Population aged 65+ (%)	192	4.6 (3.2, 10.0)	192	5.1 (3.2, 11.0)	192	5.2 (3.4, 11.4)	192	5.7 (3.4, 12.9)	192	6.0 (3.5, 13.6)	192	6.3 (3.6, 14.3)	192	6.6 (3.6, 14.9)
Total fertility rate	192	2.79 (1.86, 4.53)	192	2.57 (1.80, 4.20)	192	2.41 (1.79, 4.02)	192	2.31 (1.70, 3.77)	192	2.23 (1.64, 3.54)	192	2.16 (1.60, 3.41)	192	2.11 (1.58, 3.31)
Urban population (%)	192	53.8 (33.1, 71.8)	192	55.5 (35.8, 73.2)	192	57.5 (38.3, 74.3)	192	59.7 (39.7, 76.0)	192	60.4 (39.8, 77.0)	192	61.2 (40.6, 77.2)	192	62.0 (41.5, 77.9)

UHC, Universal Health Coverage; SCI, service coverage index; RMNCH, reproductive, maternal, newborn, and child health; ID, infectious disease; NCD, noncommunicable disease; SCA, service capacity and access; HDI, Human Development Index; GNI, gross national income.

When examining year-specific median trends by World Bank income group, a consistent pattern of higher values in higher-income groups was observed for the UHC SCI and most sub-indices, with high-income countries generally maintaining the highest medians throughout the study period, although this pattern was less distinct for the NCD sub-index (Fig 1). Median UHC SCI increased in all four income groups, rising from 23.0 to 41.5 in low-income countries and from 72.0 to 82.0 in high-income countries between 2000 and 2021. The ID sub-index also increased steadily in low- and lower-middle-income countries, with some reduction in the difference between income groups over time, whereas the SCA sub-index remained persistently low and largely flat in low-income countries (26.0 in 2000 and 22.5 in 2021).

**Fig 1:**
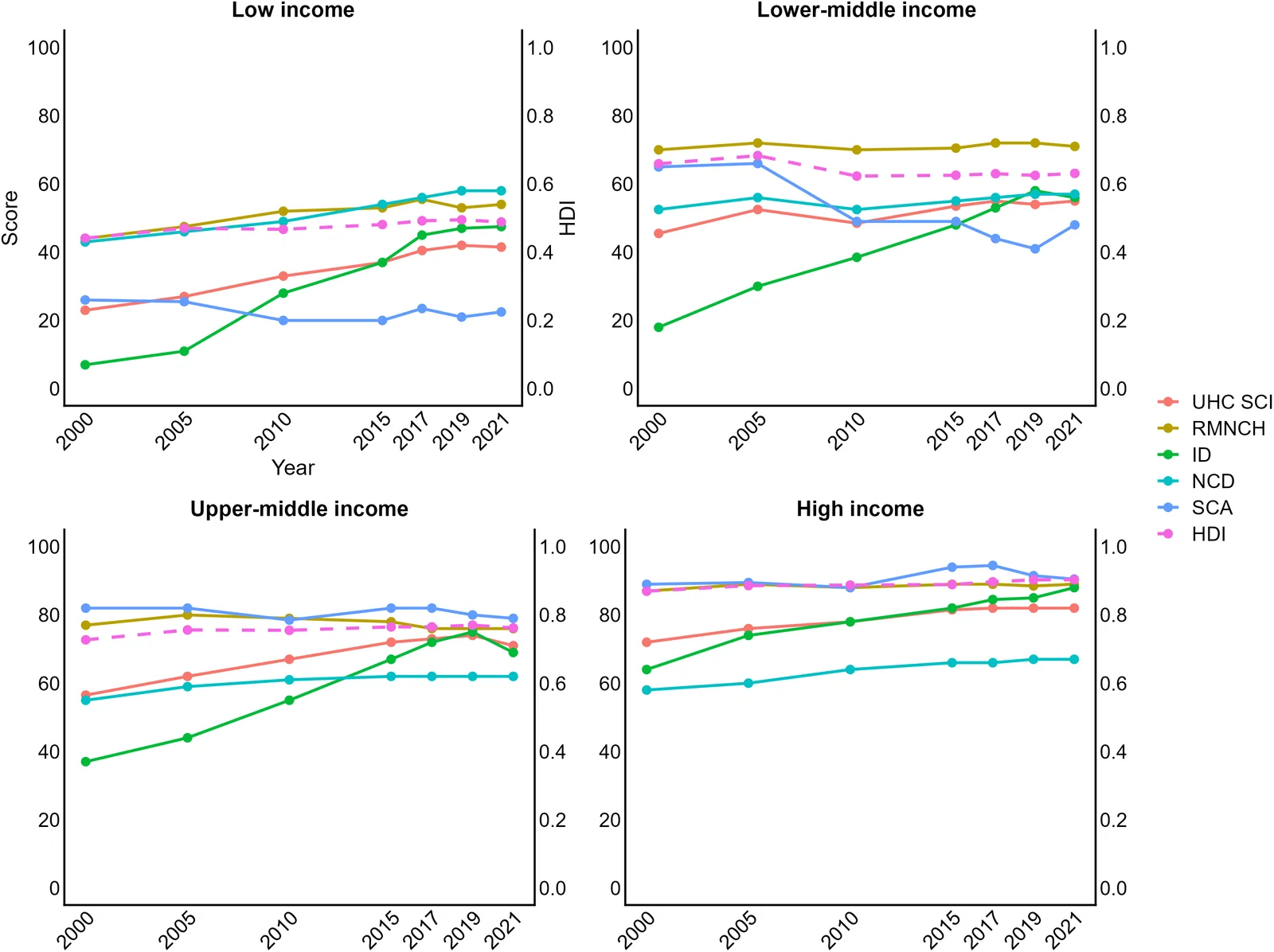
Median trends in the UHC SCI, its four sub-indices, and the HDI by World Bank income group.

Across the WHO regions, median levels and temporal patterns of the UHC SCI, HDI, and sub-indices varied (Fig 2). EUR and AMR generally maintained relatively high median levels throughout the study period; for example, the median UHC SCI increased from 69.0 to 80.0 in EUR and from 51.0 to 76.0 in AMR between 2000 and 2021. The ID sub-index increased markedly in all regions, including AFR, where it rose from 7.0 to 51.0 from a low baseline. In contrast, the SCA sub-index remained particularly low in AFR, changing only modestly from 20.0 to 27.0, whereas the NCD sub-index showed limited improvement overall: it was lowest in SEAR in 2000 (36.5), but from 2010 onward the lowest median values were observed in WPR (45.0 in 2010 and 44.5 in 2021).

**Fig 2:**
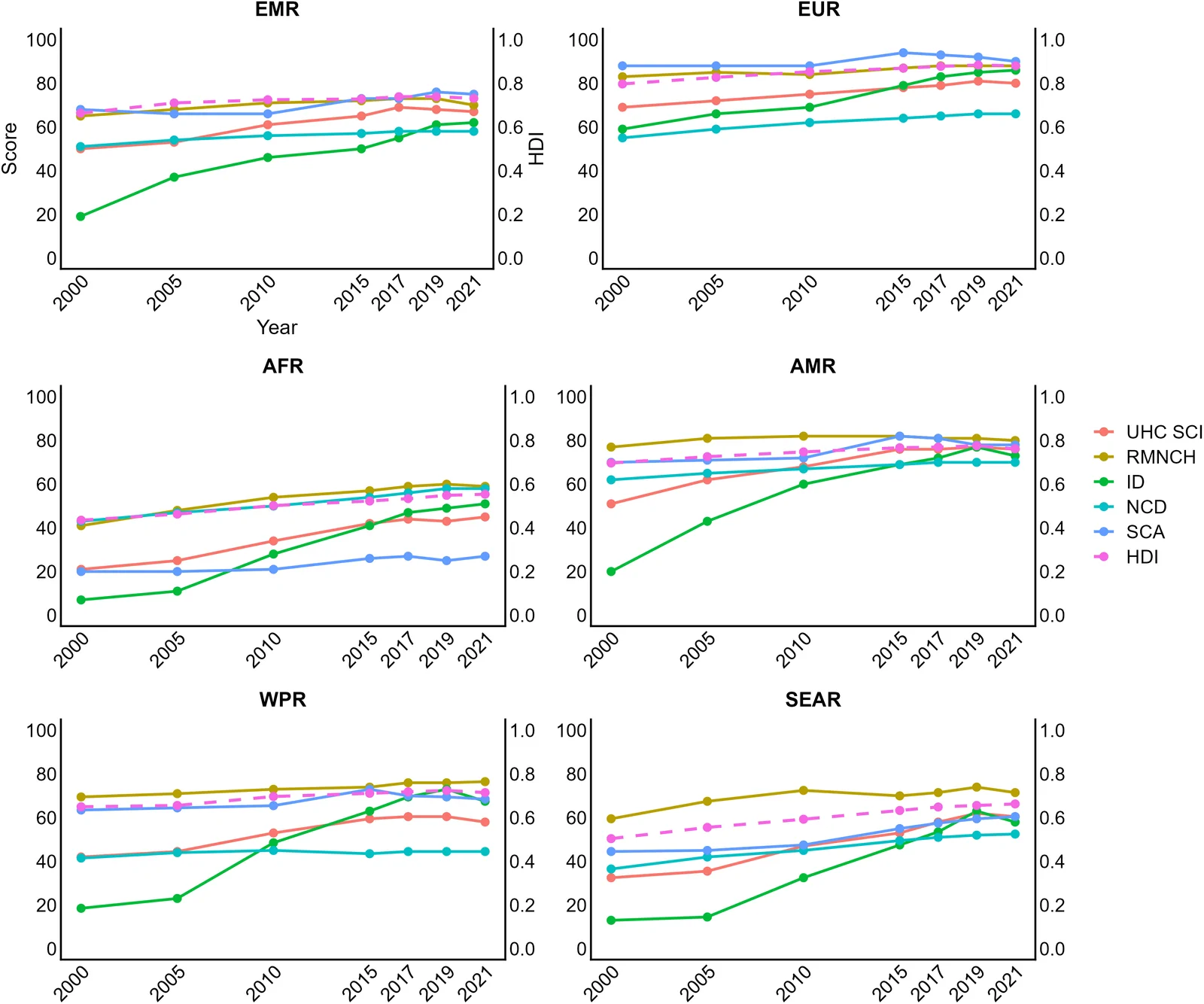
Median trends in the UHC SCI, its four sub-indices, and the HDI by WHO region.

In GEE models estimating population-averaged differences as mean ratios, the income-group analysis (reference: high-income) showed lower mean ratios in the lower-income groups in both crude and adjusted models, and most differences remained statistically significant after adjustment for covariates (Table 2). In low-income countries, the adjusted differences were most pronounced for ID, with mean levels 31% lower than those observed in high-income countries, followed by HDI (24% lower), the UHC SCI (21% lower), and SCA (20% lower). Income-group differences in RMNCH were not statistically significant in any income group; regarding NCD, the adjusted differences in the low- and lower-middle-income groups were not statistically significant. In the region-based analysis (reference: EUR), RMNCH showed little regional variation, with no region differing significantly from EUR after adjustment. In contrast, ID levels were significantly lower in all other regions than in EUR, ranging from 6% lower in AMR to 16% lower in SEAR. The UHC SCI was significantly lower in SEAR (11%), AFR (11%), and WPR (8%), whereas EMR and AMR did not differ significantly from EUR. Adjusted mean ratios indicated that HDI levels were modestly but significantly lower in all other regions than in EUR (4–10% lower). Regarding SCA, only AFR differed significantly from EUR (26% lower). Regarding NCD, only AMR (17% higher) and WPR (12% lower) differed significantly from EUR (Table 2).

**Table 2:** Crude and adjusted mean ratios of UHC service coverage and HDI by World Bank income group and WHO region.

Variable	Category	UHC SCI		RMNCH		ID		NCD		SCA		HDI	
		Crude	Adjusted	Crude	Adjusted	Crude	Adjusted	Crude	Adjusted	Crude	Adjusted	Crude	Adjusted
		mean ratio (95%CI)	mean ratio (95%CI)	mean ratio (95%CI)	mean ratio (95%CI)	mean ratio (95%CI)	mean ratio (95%CI)	mean ratio (95%CI)	mean ratio (95%CI)	mean ratio (95%CI)	mean ratio (95%CI)	mean ratio (95%CI)	mean ratio (95%CI)
Income group	High income	Ref		Ref		Ref		Ref		Ref		Ref	
	Low income	0.46 (0.43–0.49)	**0.79 (0.73–0.85)**	0.58 (0.53–0.63)	0.93 (0.86–1.00)	0.42 (0.39–0.46)	**0.69 (0.60–0.78)**	0.80 (0.76–0.84)	0.97 (0.90–1.05)	0.32 (0.27–0.39)	**0.80 (0.67–0.94)**	0.54 (0.51–0.56)	**0.76 (0.73–0.79)**
	Lower-middle income	0.67 (0.64–0.71)	**0.90 (0.86–0.94)**	0.78 (0.75–0.81)	1.00 (0.95–1.04)	0.60 (0.56–0.64)	**0.81 (0.75–0.88)**	0.85 (0.80–0.90)	0.96 (0.90–1.02)	0.61 (0.56–0.67)	0.92 (0.84–1.00)	0.72 (0.70–0.74)	**0.86 (0.84–0.89)**
	Upper-middle income	0.85 (0.82–0.88)	**0.95 (0.92–0.98)**	0.86 (0.83–0.89)	0.96 (0.93–1.00)	0.79 (0.75–0.82)	**0.92 (0.88–0.97)**	0.94 (0.90–0.98)	**0.93 (0.89–0.98)**	0.87 (0.83–0.91)	1.02 (0.96–1.07)	0.85 (0.83–0.87)	**0.94 (0.92–0.95)**
Region	EUR	Ref		Ref		Ref		Ref		Ref		Ref	
	EMR	0.75 (0.67–0.85)	0.94 (0.88–1.02)	0.78 (0.69–0.88)	0.96 (0.88–1.04)	0.69 (0.60–0.81)	**0.87 (0.78–0.96)**	0.92 (0.88–0.95)	1.01 (0.93–1.08)	0.71 (0.60–0.85)	0.97 (0.85–1.10)	0.81 (0.74–0.89)	**0.95 (0.90–0.99)**
	AFR	0.54 (0.49–0.58)	**0.89 (0.83–0.97)**	0.65 (0.60–0.71)	0.96 (0.89–1.05)	0.54 (0.49–0.59)	**0.85 (0.76–0.95)**	0.84 (0.81–0.87)	1.03 (0.95–1.12)	0.34 (0.28–0.41)	**0.74 (0.62–0.89)**	0.61 (0.58–0.65)	**0.90 (0.87–0.94)**
	AMR	0.93 (0.89–0.97)	1.01 (0.97–1.06)	0.97 (0.92–1.02)	1.04 (0.99–1.10)	0.87 (0.82–0.93)	**0.94 (0.90–0.99)**	1.10 (1.06–1.14)	**1.17 (1.11–1.23)**	0.84 (0.78–0.90)	0.93 (0.86–1.00)	0.88 (0.84–0.92)	**0.95 (0.92–0.97)**
	WPR	0.77 (0.70–0.85)	**0.92 (0.87–0.98)**	0.89 (0.83–0.95)	1.00 (0.94–1.06)	0.76 (0.69–0.85)	**0.89 (0.84–0.95)**	0.76 (0.68–0.86)	**0.88 (0.79–0.97)**	0.76 (0.67–0.86)	0.96 (0.87–1.05)	0.85 (0.79–0.91)	**0.96 (0.94–0.99)**
	SEAR	0.70 (0.62–0.78)	**0.89 (0.81–0.98)**	0.85 (0.77–0.94)	0.99 (0.90–1.10)	0.63 (0.55–0.72)	**0.84 (0.75–0.94)**	0.80 (0.72–0.90)	0.89 (0.80–1.00)	0.62 (0.50–0.76)	0.83 (0.70–1.00)	0.75 (0.69–0.82)	**0.94 (0.89–0.99)**

Bold adjusted estimates indicate statistical significance at the α = 0.05 level.

UHC, Universal Health Coverage; SCI, service coverage index; RMNCH, reproductive, maternal, newborn, and child health; ID, infectious disease; NCD, noncommunicable disease; SCA, service capacity and access; HDI, Human Development Index; AFR, African Region; AMR, Region of the Americas; EMR, Eastern Mediterranean Region; EUR, European Region; SEAR, South-East Asia Region; WPR, Western Pacific Region.

Fig 3 shows generally positive associations between human development and the UHC SCI and most sub-indices, although the magnitude of these associations differed across domains. In the covariate-adjusted models, a 0.1-unit increase in HDI was associated with a 6.8-point higher UHC SCI (95% CI 5.6–8.1). The adjusted point differences were largest for SCA (11.7 points, 95% CI 9.2–14.1), followed by ID (6.2 points, 95% CI 4.2–8.1) and RMNCH (4.9 points, 95% CI 3.2–6.7), whereas the association with NCD was smaller (2.2 points, 95% CI 0.4–4.0). When the HDI components were examined separately, all three were positively associated with the UHC SCI after adjustment, with adjusted slopes of 3.5 points (95% CI 2.4–4.6) for the GNI index, 3.4 points (95% CI 2.5–4.3) for the education index, and 4.1 points (95% CI 2.7–5.4) for the life expectancy index per 0.1-unit increase. Across the sub-indices, the GNI and life expectancy indices were positively associated with NCD after adjustment, but the estimated increases were smaller than those for most other sub-indices, whereas the education index was not associated with NCD after adjustment (Fig 3).

**Fig 3:**
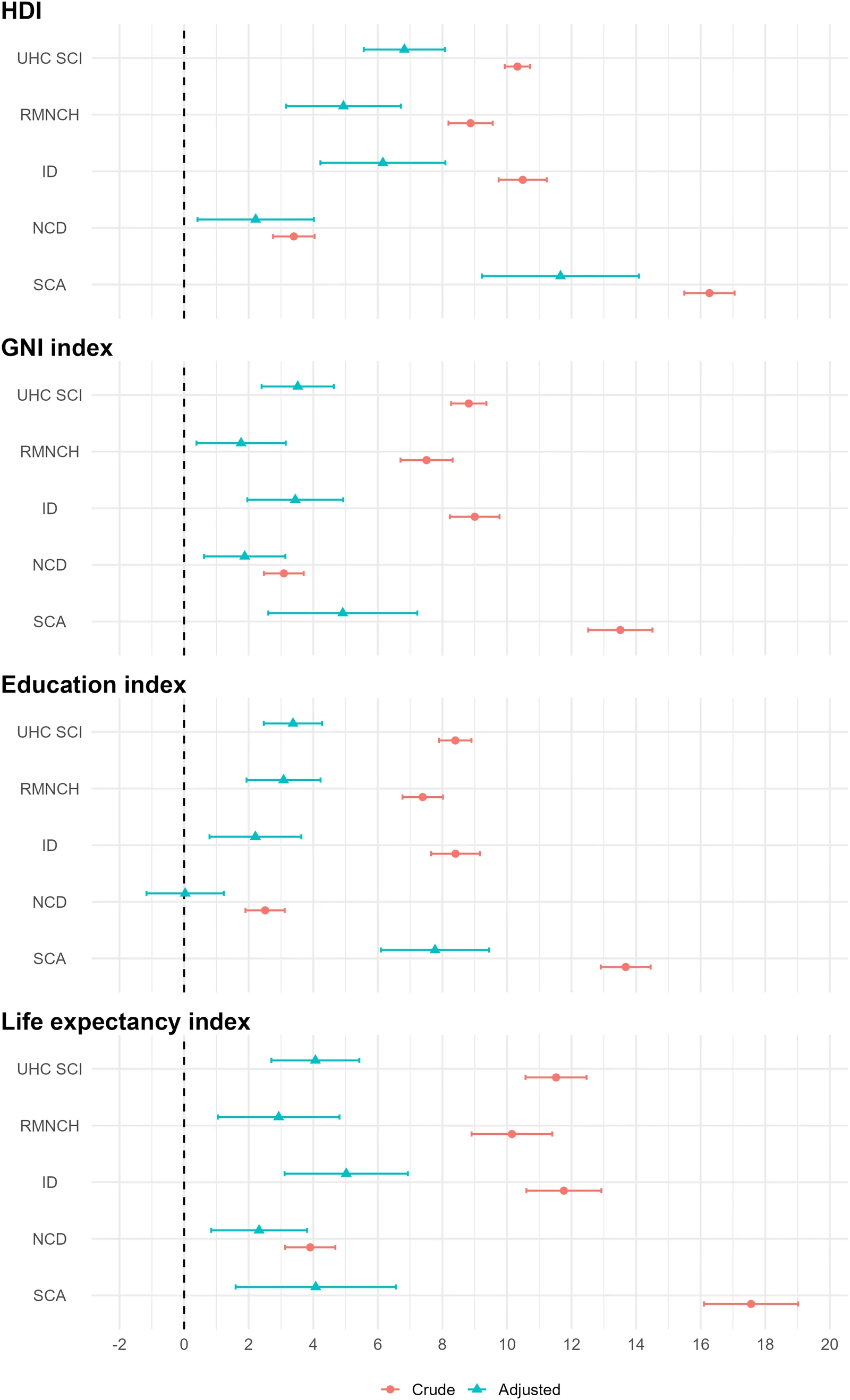
Estimated changes in UHC SCI and sub-indices per 0.1-unit increase in HDI and its component indices.

Supplementary analyses allowing the HDI-related slopes to vary by calendar year (S1–S5 Figs) showed patterns broadly consistent with the pooled estimates in Fig 3. For the UHC SCI, RMNCH, and ID, positive associations with HDI and most of its component indices were observed across study years, with some attenuation over time, particularly for ID and, to a lesser extent, RMNCH. For the adjusted HDI-related slopes, the estimates were 7.0 points (95% CI 5.7–8.3) in 2000 and 6.6 points (95% CI 5.4–7.9) in 2021 for the UHC SCI, 6.1 points (95% CI 4.2–8.0) in 2000 and 4.8 points (95% CI 3.0–6.6) in 2021 for RMNCH, and 7.1 points (95% CI 5.1–9.2) in 2000 and 5.0 points (95% CI 2.9–7.0) in 2021 for ID. Associations for NCD were consistently small with little temporal variation, from 2.5 points (95% CI 0.7–4.4) in 2000 to 1.9 points (95% CI 0.1–3.7) in 2021, whereas those for SCA increased over time, from 9.4 points (95% CI 7.0–11.9) in 2000 to 12.6 points (95% CI 10.0–15.1) in 2021.

## Discussion

In this country-level longitudinal panel study covering 2000–2021, we quantified disparities in the UHC SCI, its four domain-specific sub-indices, and the HDI across World Bank income groups and WHO regions using population-averaged longitudinal models. We also examined the associations of the UHC SCI and its four domain-specific sub-indices with the HDI and its component indices for GNI, education, and life expectancy. Overall, the UHC SCI, ID, SCA, and HDI showed persistent income-group disparities, whereas such disparities were less marked for RMNCH and less pronounced for NCD. Regional differences were generally smaller than income-group disparities after covariate adjustment, but their patterns varied across domains: SCA showed the largest differences; ID levels were consistently lower than in EUR; differences in the UHC SCI were evident in selected regions; NCD differences were mixed in direction; and RMNCH differences were limited. Higher HDI was consistently associated with better UHC performance, but the strength of this association varied across domains, being strongest for SCA, followed by ID and RMNCH, and weak for the NCD sub-index.

The patterns observed in this study are consistent with WHO and World Bank global monitoring reports, which have shown that the global population-weighted UHC SCI score increased from 45 to 68 between 2000 and 2021, but that progress slowed after 2015; the largest long-term gains were observed in ID, whereas improvements in other domains were more limited [1]. Our results are also consistent with previous reports showing that, although some convergence has occurred over time, disparities across income groups and regions persist [1]. They further align with global monitoring reports indicating that NCD coverage has shown only modest improvement and remains a relatively weak domain of service coverage [1]. We extend the existing literature by quantifying differences across income groups and regions within an integrated longitudinal multivariable framework.

Our findings on the relationship between UHC and broader human development are also consistent with previous studies. The foundational study on the UHC SCI reported that the index was strongly correlated with the HDI and remained strongly associated with life expectancy even after adjustment for income and education [2]. Other multi-country studies have similarly reported that development-related and health-system factors are closely related to progress toward UHC, highlighting the roles of development, governance, health expenditure, and workforce capacity [5,6,9,10]. In particular, previous research has shown that countries with higher HDI had significantly better health service coverage before the COVID-19 pandemic [12]. We extend existing evidence by using repeated observations over more than two decades, examining all four UHC sub-indices separately, and decomposing the HDI into its three component indices. Our findings support the view that development and UHC are closely linked and show that the strength of this relationship varies across domains.

The component-level findings should be interpreted with particular caution for the life expectancy index. Unlike the GNI and education indices, life expectancy is a component of the HDI but may be influenced by progress toward UHC through improved prevention, treatment, and access to health services. The observed associations between the life expectancy index and the UHC SCI or its sub-indices may reflect bidirectional relationships or conceptual overlap between population health and service coverage, rather than an independent contribution of life expectancy to UHC progress. Therefore, our component-level analyses should be interpreted as descriptive associations that help characterize the HDI–UHC relationship, not as a causal decomposition of the pathways linking human development and UHC.

In our study, the strongest association with HDI was observed for SCA. This pattern is plausible given that the SCA domain reflects core health system foundations, such as health infrastructure, workforce capacity, and institutional readiness, which tend to improve gradually alongside broader socioeconomic development [2,6,9]. This could be particularly relevant in lower-income settings, where limitations in hospital access and health workforce capacity remain important constraints on UHC service coverage [2,9]. The strong association between HDI and ID may likewise reflect the expansion of many infectious disease interventions over the past two decades through sustained domestic and international investments, particularly in low-income countries [1]. By contrast, the association with RMNCH was moderate. This may reflect, at least in part, the nature of the RMNCH tracer set, which includes several interventions that have long been prioritized in low- and middle-income countries and can be expanded through focused public health and primary care programs even at relatively modest levels of overall development. At the same time, this does not imply equity has been achieved, as substantial within-country inequalities in RMNCH coverage by income, education, and place of residence remain well documented [1,2,13].

In addition, the NCD sub-index showed smaller income-group disparities than the other domains and mixed regional patterns. Although HDI and some of its component indices were positively associated with NCD after adjustment, the estimated increases were smaller than those for most other sub-indices; among the HDI components, the GNI and life expectancy indices remained positively associated with NCD after adjustment, whereas the education index was not associated with NCD. At first glance, this pattern may appear counterintuitive, particularly given the relatively high baseline NCD values in the low-income group and the fact that the NCD sub-index did not follow the same ranking pattern as ID or SCA. This pattern is likely attributable, at least in part, to the way the NCD sub-index was constructed in the pre-2025 SCI data series. In the 2023 WHO and World Bank monitoring framework used in this study, the NCD sub-index included hypertension treatment, diabetes prevalence, and tobacco non-use [1]. Although hypertension treatment is closer to a service-coverage measure, diabetes prevalence and tobacco non-use do not directly capture the availability, use, or quality of NCD services. Therefore, the NCD sub-index should be interpreted as a tracer-based summary measure rather than as a comprehensive measure of chronic disease management or NCD care delivery [1]. The 2025 revision replaced the diabetes prevalence proxy with diabetes treatment coverage, but it also demonstrated that NCD sub-index estimates remain sensitive to the choice and weighting of tracer indicators, including tobacco-use reduction [4]. This measurement issue may partly explain why NCD patterns differed from those observed for SCA, ID, and RMNCH in our analyses.

### Limitations

This study has some limitations. First, the analysis was conducted at the country level and therefore could not capture within-country inequalities. This is important because substantial disparities in service coverage within countries have been documented by income, education, place of residence, and other geographic factors [1,13]. Second, the UHC SCI and some of its tracer indicators have been revised over time, and the underlying data series have also been updated. Values presented in different report editions are not directly comparable, and the 2025 revision introduced changes in tracer definitions and weighting approaches [1,4]. Some of the patterns observed in this study may change as the measurement framework continues to be revised. Third, the study period extended to 2021, and the estimates may therefore reflect the effects of the COVID-19 pandemic. The 2023 WHO and World Bank global monitoring report noted that the global UHC SCI stagnated between 2019 and 2021 and that acute disruptions in essential health service delivery during the pandemic may not have been fully captured by the annual SCI [1]. However, the 2021 SCI estimates should not be interpreted as being based entirely on services observed in calendar year 2021, because recent tracer-indicator values may include reported administrative data, survey-derived estimates, modelled estimates, imputed values, or extrapolated values. Our 2021 findings should be interpreted as reflecting the latest available benchmark estimates during the pandemic period, rather than a direct measurement of the full effect of COVID-19 on service coverage. Fourth, missing data may also have affected the generalizability of our findings. Although the UHC SCI and its four sub-indices had complete country coverage in all seven study years, HDI, its component indices, income group classification, and some covariates had missing values, particularly in the earlier years. Consequently, complete-case analyses included fewer countries in the initial study year than in later years. For example, adjusted models involving HDI included 160–161 countries in 2000, increasing to 189 countries from 2015 onward. Across the main adjusted analyses, complete-case samples generally included 1,278–1,323 country-year observations overall. Missingness may be systematically related to country characteristics and may be more common among small states, countries with weaker statistical systems, and fragile or conflict-affected settings. Therefore, complete-case analyses may underrepresent some settings where UHC challenges are substantial, particularly in earlier years.

## Conclusion

In conclusion, this study reported that higher levels of human development were associated with better UHC service coverage overall, although the magnitude of this association varied across domains. The association was strongest for SCA and ID, moderate for RMNCH, and weak for the NCD sub-index. This could reflect limitations in how the NCD domain is measured within the SCI framework. Persistent disparities across income groups, together with the markedly lower adjusted SCA level in the African Region compared with the European Region, suggest that progress toward UHC remains closely tied to broader development conditions and to regional differences in health-system capacity. These findings may help policymakers interpret the UHC SCI and its sub-indices in the context of their country’s broader development trajectory.

## Supporting information

S1 Fig Year-specific changes in UHC SCI per 0.1-unit increase in HDI and its component indices. (TIFF)

S2 Fig Year-specific changes in the RMNCH sub-index per 0.1-unit increase in HDI and its component indices. (TIFF)

S3 Fig Year-specific changes in the ID sub-index per 0.1-unit increase in HDI and its component indices. (TIFF)

S4 Fig Year-specific changes in the NCD sub-index per 0.1-unit increase in HDI and its component indices. (TIFF)

S5 Fig Year-specific changes in the SCA sub-index per 0.1-unit increase in HDI and its component indices. (TIFF)
