# Supplementary material for: Associations between the universal health coverage service coverage index and the human development index: A country-level longitudinal panel study
Source: PLOS Glob Public Health. 2026 Oct 28;6(10):e0007328. doi: 10.1371/journal.pgph.0007328 (PMC13716186; doi:10.1371/journal.pgph.0007328)
Supplement: Supplementary file 1 [file pgph.0007328.s007.docx]

July 16, 2026

Paolo Angelo Cortesi, PhD

Academic Editor

*PLOS Global Public Health*

Dear Dr Cortesi,

Thank you for the careful consideration of our manuscript and for the constructive comments from the editor and reviewers. We have revised our manuscript in response to the reviewers’ comments. Please find below our point-by-point responses to each comment. We indicate the corresponding changes in the revised manuscript by their page and line numbers below.

**Correction identified during revision:**

While preparing this revision, we identified that the analysis code for Table 2 had inadvertently omitted the year fixed effects described in the Statistical analysis section for these population-averaged models. We corrected the code so that both the unadjusted and adjusted models now consistently include year fixed effects, and we updated Table 2 and the corresponding text in the Results section (Page 16, Lines 247-261) and Discussion section (Page 19, Lines 304-308; Page 21, Lines 360-361) accordingly. The main conclusions of the study are unchanged: higher human development remained associated with better UHC service coverage; the association was strongest for SCA and ID and weakest for NCD; and persistent income-group disparities remained for the UHC SCI, ID, SCA, and HDI. The principal substantive change was in the region-based analysis: after the correction, mean ID sub-index levels were significantly lower in all other regions than in the European Region, rather than showing no significant regional differences. The statistical significance of several other income-group and regional comparisons also changed, but the overall interpretation of the findings remained unchanged. The updated analysis code is available in the GitHub repository cited in the Data Availability Statement.

**Journal Requirements:**

**Requirement 1:**

We have noticed that you have uploaded Supporting Information files, but you have not included a list of legends. Please add a full list of legends for your Supporting Information files after the references list.

**Response 1:**

We thank the journal staff for noting this issue. The legends for the Supporting Information files were included in the original manuscript after the reference list. To make them easier to identify, we have now placed them under a separate “Supporting Information” heading on a new page after the references (Page 29, Lines 513-523).

**Requirement 2:**

We note that your Data Availability Statement is currently as follows: "All accompanying code and data to generate all tables and figures in the main manuscript and Supporting Information are available on GitHub: https://github.com/AH1026/UHC-HDI_2026"

**Response 2:**

We confirm that the submission contains the minimal dataset required to replicate all findings reported in the manuscript. The country-year analytic dataset, the values underlying all tables and figures in the main manuscript and Supporting Information, and the R code used for the analyses are publicly available in the GitHub repository identified in the Data Availability Statement.

**Requirement 3:**

Any changes to the reference list should be mentioned in the rebuttal letter that accompanies your revised manuscript. If you need to cite a retracted article, indicate the article’s retracted status in the References list and also include a citation and full reference for the retraction notice.

**Response 3:**

We reviewed the reference list and made two changes. We updated Reference [4] from the advance proof/conference edition to the final published version of the 2025 Global Monitoring Report (Page 25, Lines 440-444). We also corrected the issue number for Reference [10] from 15(3) to 15(2), without changing the cited article, DOI, or page range (Page 26, Lines 462-464). No other changes were made to the reference list, and no retracted articles are cited.

**To Reviewer #1:**

**Comment 1:**

The manuscript should address COVID-19. Since the study period ends in 2021, the global pandemic may have affected health service coverage, service capacity and access, and HDI trends. This should be acknowledged in the paper.

**Response 1:**

We thank the reviewer for this important comment. We agree that the COVID-19 pandemic should be addressed because our study period extends to 2021. We have added text to the Limitations section noting that the pandemic may have affected health service coverage, service capacity and access, and HDI trends (Pages 22-23, Lines 390-399). We also clarified that the 2021 UHC SCI estimates should not be interpreted as fully observed calendar-year 2021 service coverage for all countries and tracer indicators. According to the 2023 WHO and World Bank global monitoring report, the global UHC SCI stagnated between 2019 and 2021, but acute disruptions in essential health service delivery may not have been fully captured by the annual SCI. The report also notes that recent tracer-indicator values may include reported, survey-derived, modelled, imputed, or extrapolated values. We therefore interpret the 2021 estimates as benchmark estimates during the pandemic period rather than as direct estimates of the causal impact of COVID-19.

**Comment 2:**

The authors should strengthen the discussion of life expectancy as an HDI component. Life expectancy is used as part of the explanatory variable, but it may also be affected by UHC progress. This creates some overlap in interpretation, and should be explained more clearly as a limitation.

**Response 2:**

We agree with the reviewer that the life expectancy index requires careful interpretation because it is both a component of the HDI and a potential downstream consequence of UHC progress. Although the original manuscript noted this issue in the Variables section, we have expanded the Discussion to explain that associations involving the life expectancy index may reflect bidirectional relationships or conceptual overlap between population health and service coverage (Pages 20-21, Lines 335-344).

**Comment 3:**

The manuscript notes missing data especially in initial years. The authors should explain how this missingness may affect the results, especially because countries with missing data may be poorer, conflict affected, smaller, or have weaker data systems. If such countries are left out of some analyses, the findings may not fully reflect countries where UHC challenges are greatest

**Response 3:**

We thank the reviewer for this important comment. We agree that missing data may affect the interpretation and generalizability of the findings. We have expanded the Limitations section to discuss this issue more explicitly (Page 23, Lines 399-410). In our dataset, the UHC SCI and its four sub-indices had complete country coverage in all seven study years, while HDI, its component indices, income group classification, and some covariates had missing values, particularly in earlier years. Although the complete-case samples for the main adjusted analyses generally included 1,277–1,323 country-year observations overall, country coverage was lower in the earliest study year. For example, the adjusted models involving HDI included 160–161 countries in 2000, increasing to 189 countries from 2015 onward. We now clarify that missingness may be systematically related to country characteristics and may be more common among small states, countries with weaker statistical systems, and fragile or conflict-affected settings. We therefore acknowledge that complete-case analyses may underrepresent some countries where UHC challenges are substantial, particularly in earlier years.

**Comment 4:**

The selected covariates are reasonable but the rationale for including them should be explained and clarify why health expenditure was not included since it was discussed in the introduction as an important factor associated with UHC

**Response 4:**

We thank the reviewer for this helpful comment. We agree that the rationale for covariate selection should be stated more clearly, particularly because health expenditure was discussed in the Introduction as an important factor associated with UHC. We have revised the Statistical analysis section to clarify that adjusted models included covariates selected a priori to account for governance, demographic structure, population health needs, and service-access context (Pages 9-10, Lines 170-176).

We also clarified why health expenditure was not included in the primary adjustment set (Page 10, Lines 176-180). Although previous studies have shown that health expenditure, including domestic general government health expenditure, is associated with UHC service coverage, we considered health expenditure to be closely linked to income and broader socioeconomic development and to potentially represent a downstream financing mechanism through which public-sector capacity and policy priorities affect service coverage. We therefore did not treat health expenditure as a baseline confounder for our primary objective.

As an additional exploratory check to inform this decision, we examined cross-sectional rank correlations in the latest study year, 2021, between per-capita health expenditure variables from the WHO Global Health Expenditure Database and the main development variables in our analytic dataset. Current health expenditure per capita was highly correlated with World Bank income group treated as an ordinal variable (Spearman’s ρ = 0.94, 95% CI 0.92–0.95), HDI (ρ = 0.94, 95% CI 0.92–0.96), and the GNI index (ρ = 0.94, 95% CI 0.91–0.95). Domestic general government health expenditure per capita showed similarly high correlations with income group (ρ = 0.93, 95% CI 0.91–0.95), HDI (ρ = 0.94, 95% CI 0.92–0.95), and the GNI index (ρ = 0.94, 95% CI 0.92–0.96). These checks further supported our decision not to include health expenditure in the primary adjustment set because doing so could introduce conceptual overlap and potential overadjustment.

**Comment 5:**

The limitations of the NCD sub index may need to be flagged earlier in the manuscript, not only in the discussion. Since the pre 2025 NCD sub-index includes proxy indicators that may not directly measure actual NCD service coverage, readers should be reminded to interpret the NCD findings with that context

**Response 5:**

We thank the reviewer for this important comment. We agree that the limitations of the NCD sub-index should be flagged earlier in the manuscript. In the original submission, we discussed this issue mainly in the Discussion. We have now added text to the Introduction to alert readers that the NCD findings should be interpreted in light of the tracer indicators used in the pre-2025 SCI data series. Specifically, we clarified that, in the 2023 WHO and World Bank monitoring framework, the NCD sub-index included hypertension treatment, diabetes prevalence, and tobacco non-use, and that it should be interpreted as a tracer-based summary measure rather than as a comprehensive measure of NCD service coverage, chronic disease management, or quality of NCD care (Page 4, Lines 64-70).

We also revised the Discussion to describe the measurement limitations of the NCD sub-index more accurately and cautiously. In particular, we clarified that hypertension treatment is closer to a service-coverage measure, whereas diabetes prevalence and tobacco non-use do not directly capture the availability, use, or quality of NCD services. We further explained that the 2025 SCI revision replaced the diabetes prevalence proxy with diabetes treatment coverage, but also highlighted that NCD sub-index estimates remain sensitive to the choice and weighting of tracer indicators, including tobacco-use reduction. We therefore describe the 2025 revision as illustrating ongoing measurement issues rather than as fully resolving the limitations of the NCD sub-index (Page 22, Lines 367-379).

In addition, we updated our description of the 2025 Global Monitoring Report. At the time of our original submission, the report was available as an advance proof/conference edition, and the document stated that its content was not final. Since the final version has now been published, we updated Reference [4] to cite the final report and revised the relevant manuscript text accordingly (Page 4, Lines 63-64).

**To Reviewer #2:**

**Comment 1:**

The manuscript was well written and easy to follow. Data used in this study is also publicly available making it easily accessible. The analysis methods were adequately performed and explained and the conlcusions made were based on the data presented.

**Response 1:**

We thank the reviewer for the positive assessment of our manuscript. We are pleased that the reviewer found the manuscript well written, the data publicly available, the methods adequately performed and explained, and the conclusions supported by the presented data. We have revised the manuscript in response to Reviewer #1 and the journal requirements.

Sincerely,

Yasuhiro Nakanishi

Submission ID: PGPH-D-26-00964
