# Supplementary material for: Associations between the universal health coverage service coverage index and the human development index: A country-level longitudinal panel study
Source: PLOS Glob Public Health. 2026 Oct 28;6(10):e0007328. doi: 10.1371/journal.pgph.0007328 (PMC13716186; doi:10.1371/journal.pgph.0007328)
Supplement: Peer review history

# Peer review: Decision Letter 0

**DOI:** 10.1371/journal.pgph.0007328.r001

**Authors:** Paolo Angelo Cortesi

7 Jul 2026

PGPH-D-26-00964

Associations between the universal health coverage service coverage index and the human development index: a country-level longitudinal panel study

PLOS Global Public Health

Dear Dr. Nakanishi,

Thank you for submitting your manuscript to PLOS Global Public Health. After careful consideration, we feel that it has merit but does not fully meet PLOS Global Public Health’s publication criteria as it currently stands. Therefore, we invite you to submit a revised version of the manuscript that addresses the points raised during the review process.

Please submit your revised manuscript by Aug 06 2026 11:59PM. If you will need more time than this to complete your revisions, please reply to this message or contact the journal office at globalpubhealth@plos.org. When you're ready to submit your revision, log on to https://www.editorialmanager.com/pgph/ and select the 'Submissions Needing Revision' folder to locate your manuscript file.

Please include the following items when submitting your revised manuscript:

- A letter that responds to each point raised by the editor and reviewer(s). You should upload this letter as a separate file labeled 'Response to Reviewers'.
- A marked-up copy of your manuscript that highlights changes made to the original version. You should upload this as a separate file labeled 'Revised Manuscript with Track Changes'.
- An unmarked version of your revised paper without tracked changes. You should upload this as a separate file labeled 'Manuscript'.

Guidelines for resubmitting your figure files are available below the reviewer comments at the end of this letter.

As the corresponding author, your ORCID iD is verified in the submission system and will appear in the published article. PLOS supports the use of ORCID, and we encourage all coauthors to register for an ORCID iD and use it as well. Please encourage your coauthors to verify their ORCID iD within the submission system before final acceptance, as unverified ORCID iDs will not appear in the published article. *Only*  the individual author can complete the verification step; PLOS staff *cannot*  verify ORCID iDs on behalf of authors.

We look forward to receiving your revised manuscript.

Kind regards,

Paolo Angelo Cortesi, PhD

Academic Editor

PLOS Global Public Health

Journal Requirements:

1. We have noticed that you have uploaded Supporting Information files, but you have not included a list of legends. Please add a full list of legends for your Supporting Information files after the references list.

2. We note that your Data Availability Statement is currently as follows: "All accompanying code and data to generate all tables and figures in the main manuscript and Supporting Information are available on GitHub: https://github.com/AH1026/UHC-HDI_2026"

Please confirm at this time whether or not your submission contains all raw data required to replicate the results of your study. Authors must share the “minimal data set” for their submission. PLOS defines the minimal data set to consist of the data required to replicate all study findings reported in the article, as well as related metadata and methods (https://journals.plos.org/plosone/s/data-availability#loc-minimal-data-set-definition).

For example, authors should submit the following data:

- The values behind the means, standard deviations and other measures reported;

- The values used to build graphs;

- The points extracted from images for analysis.

Authors do not need to submit their entire data set if only a portion of the data was used in the reported study.

If your submission does not contain these data, please either upload them as Supporting Information files or deposit them to a stable, public repository and provide us with the relevant URLs, DOIs, or accession numbers. For a list of recommended repositories, please see https://journals.plos.org/plosone/s/recommended-repositories.

If there are ethical or legal restrictions on sharing a de-identified data set, please explain them in detail (e.g., data contain potentially sensitive information, data are owned by a third-party organization, etc.) and who has imposed them (e.g., an ethics committee). Please also provide contact information for a data access committee, ethics committee, or other institutional body to which data requests may be sent. If data are owned by a third party, please indicate how others may request data access.

If the reviewer comments include a recommendation to cite specific previously published works, please review and evaluate these publications to determine whether they are relevant and should be cited. There is no requirement to cite these works unless the editor has indicated otherwise.

Please review your reference list to ensure that it is complete and correct. If you have cited papers that have been retracted, please include the rationale for doing so in the manuscript text, or remove these references and replace them with relevant current references. Any changes to the reference list should be mentioned in the rebuttal letter that accompanies your revised manuscript. If you need to cite a retracted article, indicate the article’s retracted status in the References list and also include a citation and full reference for the retraction notice.

Additional Editor Comments (if provided):

[Note: HTML markup is below. Please do not edit.]

Reviewers' comments:

Reviewer's Responses to Questions

**Comments to the Author**

1. Does this manuscript meet PLOS Global Public Health’s publication criteria?

Reviewer #1: Partly

Reviewer #2: Yes

**********

2. Has the statistical analysis been performed appropriately and rigorously?

Reviewer #1: Yes

Reviewer #2: Yes

**********

3. Have the authors made all data underlying the findings in their manuscript fully available (please refer to the Data Availability Statement at the start of the manuscript PDF file)?

The PLOS Data policy

Reviewer #1: Yes

Reviewer #2: Yes

**********

4. Is the manuscript presented in an intelligible fashion and written in standard English?

Reviewer #1: Yes

Reviewer #2: Yes

**********

Reviewer #1: I believe this is a relevant and well written manuscript. The appropriate methods were used and the main conclusions are broadly supported by the results. However, I recommend minor revision before acceptance

1. The manuscript should address COVID-19. Since the study period ends in 2021, the global pandemic may have affected health service coverage, service capacity and access, and HDI trends. This should be acknowledged in the paper.

2. The authors should strengthen the discussion of life expectancy as an HDI component. Life expectancy is used as part of the explanatory variable, but it may also be affected by UHC progress. This creates some overlap in interpretation, and should be explained more clearly as a limitation.

3. The manuscript notes missing data especially in initial years. The authors should explain how this missingness may affect the results, especially because countries with missing data may be poorer, conflict affected, smaller, or have weaker data systems. If such countries are left out of some analyses, the findings may not fully reflect countries where UHC challenges are greatest

4. The selected covariates are reasonable but the rationale for including them should be explained and clarify why health expenditure was not included since it was discussed in the introduction as an important factor associated with UHC

5. The limitations of the NCD sub index may need to be flagged earlier in the manuscript, not only in the discussion. Since the pre 2025 NCD sub-index includes proxy indicators that may not directly measure actual NCD service coverage, readers should be reminded to interpret the NCD findings with that context

This is a useful and potentially publishable manuscript, but would be stronger if the authors addressed these issues

Reviewer #2: The manuscript was well written and easy to follow. Data used in this study is also publicly available making it easily accessible. The analysis methods were adequately performed and explained and the conlcusions made were based on the data presented.

**********

what does this mean?). If published, this will include your full peer review and any attached files.

**Do you want your identity to be public for this peer review?**  If you choose “no”, your identity will remain anonymous but your review may still be made public.

For information about this choice, including consent withdrawal, please see our Privacy Policy

Reviewer #1: **Yes:**  Winifred Addo-Cobbiah

Reviewer #2: **Yes:**  Sheillah Ansiima

**********

[NOTE: If reviewer comments were submitted as an attachment file, they will be attached to this email and accessible via the submission site. Please log into your account, locate the manuscript record, and check for the action link "View Attachments". If this link does not appear, there are no attachment files.]

# Author comment: Author response to Decision Letter 1

**DOI:** 10.1371/journal.pgph.0007328.r002

16 Jul 2026

Attachment Submitted filename: Response_to_Reviewers_20260713.docx

# Peer review: Decision Letter 1

**DOI:** 10.1371/journal.pgph.0007328.r003

**Authors:** Paolo Angelo Cortesi

26 Aug 2026

Associations between the universal health coverage service coverage index and the human development index: a country-level longitudinal panel study

PGPH-D-26-00964R1

Dear Dr Nakanishi,

We are pleased to inform you that your manuscript 'Associations between the universal health coverage service coverage index and the human development index: a country-level longitudinal panel study' has been provisionally accepted for publication in PLOS Global Public Health.

Before your manuscript can be formally accepted you will need to complete some formatting changes, which you will receive in a follow up email. A member of our team will be in touch with a set of requests.

Please note that your manuscript will not be scheduled for publication until you have made the required changes, so a swift response is appreciated.

IMPORTANT: The editorial review process is now complete. PLOS will only permit corrections to spelling, formatting or significant scientific errors from this point onwards. Requests for major changes, or any which affect the scientific understanding of your work, will cause delays to the publication date of your manuscript.

If your institution or institutions have a press office, please notify them about your upcoming paper to help maximize its impact. If they'll be preparing press materials, please inform our press team as soon as possible -- no later than 48 hours after receiving the formal acceptance. Your manuscript will remain under strict press embargo until 2 pm Eastern Time on the date of publication. For more information, please contact globalpubhealth@plos.org.

Thank you again for supporting Open Access publishing; we are looking forward to publishing your work in PLOS Global Public Health.

Best regards,

Paolo Angelo Cortesi, PhD

Academic Editor

PLOS Global Public Health

***********************************************************

Reviewer Comments (if any, and for reference):

Reviewer's Responses to Questions

**Comments to the Author**

Reviewer #1: All comments have been addressed

Reviewer #2: All comments have been addressed

**********

publication criteria?

Reviewer #1: Yes

Reviewer #2: Yes

**********

3. Has the statistical analysis been performed appropriately and rigorously?

Reviewer #1: Yes

Reviewer #2: Yes

**********

4. Have the authors made all data underlying the findings in their manuscript fully available (please refer to the Data Availability Statement at the start of the manuscript PDF file)?

The PLOS Data policy

Reviewer #1: Yes

Reviewer #2: Yes

**********

5. Is the manuscript presented in an intelligible fashion and written in standard English?

Reviewer #1: Yes

Reviewer #2: Yes

**********

Reviewer #1: Thank you for the revision. Very thorough. All my previous concerns have been addressed adequately

I also particularly liked that the authors proactively disclosed and corrected a coding error in Table 2 during revision, in which year fixed effects had inadvertently been omitted from the population-averaged models

I am satisfied that the manuscript has been meaningfully improved in response to the concerns raised so I recommend acceptance

Reviewer #2: I would like to thank the authors for the careful consideration to all the comments raised by the journal and the other reviewer. I can confirm that these comments were sufficiently addressed. The legends on the supporting information make it easier to inteprete the graphs.

**********

what does this mean?). If published, this will include your full peer review and any attached files.

**Do you want your identity to be public for this peer review?**  If you choose “no”, your identity will remain anonymous but your review may still be made public.

For information about this choice, including consent withdrawal, please see our Privacy Policy

Reviewer #1: **Yes:**  Winifred Addo-Cobbiah

Reviewer #2: **Yes:**  Sheillah Ansiima

**********
